# Making sense of fidelity: young Africans’ cross-national and longitudinal representations of fidelity and infidelity in their HIV-related creative narratives, 1997–2014

**DOI:** 10.1080/17290376.2021.1950042

**Published:** 2021-07-06

**Authors:** Robyn Singleton, Manon Billaud, Haley McLeod, Georges Tiendrebeogo, Fatim Dia, Chris Obong’o, Siphiwe Nkambule-Vilakati, Benjamin Mbakwem, Gaelle Sabben, Kate Winskell

**Affiliations:** a Senior Research and Evaluation Strategist, Banyan Communications, Atlanta, GA, USA; b Behavioral Sciences and Health Education Department, Rollins School of Public Health at Emory University, Atlanta, GA, USA; c Hubert Department of Global Health, Rollins School of Public Health at Emory University, Atlanta, GA, USA; d Laafi Consulting, Ouagadougou, Burkina Faso; e PATH, Nairobi, Kenya; f Super Buddies Club, Mbabane, Swaziland; g Community and Youth Development Initiatives, Owerri, Nigeria

**Keywords:** Fidelity, multiple concurrent sexual partners, HIV, sub-Saharan Africa, young people, qualitative

## Abstract

Mutual fidelity and partner reduction have been identified as key behavioural strategies to prevent HIV transmission in sub-Saharan Africa, particularly following recognition of the role that multiple concurrent sexual partnerships play in driving generalised HIV epidemics. We analysed social representations of fidelity and infidelity in a sample of 1,343 narratives about HIV written by young Africans between 1997 and 2014. The narratives were written at four different time points (1997, 2005, 2008, 2014) by authors aged 10–24 in urban and rural areas of Senegal, Burkina Faso, South-east Nigeria, Kenya and Eswatini. We combined three analytical approaches: descriptive statistics of quantifiable characteristics of the narratives, thematic data analysis and a narrative-based approach. In the sample, fidelity is often promoted as the ideal by narrators, peers and romantic partners, in line with broader discourses around HIV prevention, romantic relationships, familial obligations, and religious and moral imperatives. However, mutual fidelity is rarely modelled in the narratives and representations of combining methods to prevent HIV from entering relationships via infidelity are uncommon. Representations of fidelity reflect loss-framed fear arousal techniques that perpetuate HIV-related stigma. Narrative-based approaches that facilitate skills-building, critical reflection and address stigma can better address fidelity and partner reduction.

## Introduction

Mutual fidelity and partner reduction have been identified as key behavioural strategies to prevent HIV transmission. The term ‘fidelity’ can refer to ‘lifelong monogamy, serial monogamy, faithfulness within a polygamous marriage or an overall reduction in the number of one’s casual sexual partners’ (Cohen, 2004, p. 11). The importance of fidelity and partner reduction as HIV-related behavioural practices was cast in a new light in the mid-2000s by growing understanding of the role that multiple concurrent sexual partnerships (MCPs) play in driving HIV transmission (Halperin & Epstein, 2004). While MCPs have been variably defined, in general the term refers to cases where an individual has overlapping sexual relationships with more than one partner. Different from serial or sequential monogamous partnerships, in which an individual has multiple partners over a period of time but none overlap, MCPs are epidemiologically important due to their role in spreading HIV throughout a sexual network during the early, acute phase of infection in which an individual’s HIV viral load is extremely high and the likelihood of transmission to others is increased. The longer the duration of overlapping partnerships, the greater the chances of HIV transmission within the network if one partner is or becomes infected, in part due to the negative influence that increased familiarity within a couple (and by extension, trust) has on consistent condom use (Chimbiri, 2007). Generalised epidemics, such as those seen in sub-Saharan Africa, are typically characterised by high rates of MCPs (Mah & Halperin, 2010).

Fidelity and partner reduction have long been promoted in HIV prevention efforts. Uganda in particular is credited with successfully promoting partner reduction, among other strategies, resulting in a substantial decline in HIV incidence for a time (Green, Halperin, Nantulya, & Hogle, 2006). Drawing on Uganda’s success, in the mid-2000s the US President’s Emergency Plan for AIDS Relief (PEPFAR) promoted fidelity and partner reduction as one of the key strategies within their ‘ABC’ approach, i.e. (A): abstinence or delay of sexual activity; (B) be faithful (including partner reduction and avoiding high risk partners); and (C) condom use, particularly for high risk sex (Parkhurst, 2012). During this same time period, epidemiological understanding of the role of MCPs in driving HIV transmission in generalised epidemics was growing (Halperin & Epstein, 2004; Morris & Kretzschmar, 1997).

Following increased availability of PEPFAR funds to promote the ‘ABCs’, fidelity promotion often became coupled with (and, some argue, secondary to) promotion of abstinence until marriage. Cohen (2004) even went so far as to label fidelity as the ‘forgotten middle child’ in the culture wars between conservative proponents of abstinence-only education and liberal, scientific and AIDS activist communities advocating for condom promotion. Critics of an ABC approach that emphasises abstinence until and fidelity within marriage argue that this approach was motivated by religiously driven political forces rather than based on scientific and culturally responsive evidence (Buse, Hildebrand, & Hawkes, 2016; Collins, Coates, & Curran, 2008; Parkhurst, 2012). Specifically, critics argue that the individualistic nature of fidelity promotion within the ABC approach obscures the social and structural drivers of MCPs, in particular the influence of unequal gender or sexual norms (LeClerc-Madlala, 2009); varying religious orientations towards monogamy and polygyny; transactional sex (Swidler & Watkins, 2007); economic trends that facilitate labor-driven migration (Epstein, 2007); and the role of modernity in influencing relationships (Hirsch et al., 2009). By ignoring these drivers of MCPs, fidelity within marriage promotion furthered moralistic discourse that linked HIV with ‘immoral’ sexual behaviour that ultimately contributed to people hiding infidelity rather than incorporating additional methods to address the risks of MCPs (Hirsch et al., 2009).

Upon the change in U.S. presidential administrations in 2008 and amid growing evidence against the ABC approach, HIV prevention efforts increasingly addressed MCPs rather than restricting emphasis to solely fidelity within marriage. For example, the multi-country OneLove campaign in Southern Africa sought to address the elevated HIV risk that MCPs carry to married and unmarried individuals, in addition to addressing the emotional and material dimensions of MCPs and modelling mutual fidelity as a way to enhance relationship wellbeing (Scheepers, 2013). Despite these adaptations in framing and approach, there are calls to abandon fidelity promotion, in part due to its association with the promotion of abstinence until marriage. For example, the United Nations voted to abandon both abstinence and fidelity-based HIV prevention efforts (Buse et al., 2016), citing a 2015 study of almost 500,000 people in sub-Saharan Africa that indicated that PEPFAR funding of abstinence and fidelity education did not lead to decreased HIV incidence (Lo, Lowe, & Bendavid, 2016).

There is substantial evidence in favour of comprehensive sexual education over abstinence-only education, in which fidelity within relationships is a component, in terms of enhancing young people’s knowledge and skills to navigate the complex terrain of sexual relationships (Denford, Abraham, Campbell, & Busse, 2017; Fonner, Armstrong, Kennedy, O'Reilly, & Sweat, 2014; Woog & Kågesten, 2017). Given the epidemiological role of MCPs in HIV risk, addressing partner reduction and mutual fidelity should still have a role in broader HIV prevention efforts. To achieve their aims, fidelity and partner reduction communication must account for how young Africans make sense of these concepts in the context of their social, emotional, material and health-related needs, aspirations and fears.

This paper seeks to contribute to HIV prevention and communication by providing insight into young Africans’ sense-making of fidelity amidst the evolving HIV prevention debates, campaigns and related discourses. To achieve this goal, we analyze young Africans’ creative narratives about HIV to explore social representations (Moscovici, 1981) of fidelity across five countries in sub-Saharan Africa between 1997 and 2014. Narratives are a source of insight into how people make sense of the world and how they communicate those understandings to others (Bruner, 1990). In this study, they allow cross-national and longitudinal insight into the cultural meanings and resources available to young people within and across the five countries as they seek to make sense of fidelity in the evolving context of the HIV epidemic and corresponding response. The overarching purpose of this analysis is to inform communication interventions that address fidelity as part of multi-level efforts to prevent HIV.

## Methods

### Study context

The research described in this paper is part of an ongoing five-country longitudinal study of young Africans’ social representations of HIV and AIDS (Winskell, Singleton, & Sabben, 2018). The five African countries included in the sample (Senegal, Burkina Faso, South-East Nigeria, Kenya and Eswatini) vary in their socio-cultural and epidemiological profiles. While Senegal is overwhelmingly Muslim, religious affiliation among the Burkinabè is more diverse, and the Igbo-speaking South-East Nigeria is, like Kenya and Eswatini, overwhelmingly Christian. Based on retrospectively adjusted UNAIDS figures (UNAIDS, 2018), HIV prevalence peaked at various time points across the five countries: 3.4% in Burkina Faso (1997); 10.8% in Kenya (1997); 3.8% in Nigeria (2004/2005); 0.6% in Senegal (2005); and 27.4% in Eswatini (2012). Kenya and Nigeria began receiving PEPFAR funding in 2004, and Eswatini in 2007.

### Study sample

We analyzed de-identified narratives about HIV submitted to scriptwriting competitions by young people aged 10–24 at four discrete time points: 1997, 2005, 2008 and 2014. The competitions were coordinated internationally by the non-profit organisation Global Dialogues (www.globaldialogues.org). Contest participants were invited to contribute a creative idea for a short film about HIV. We stratified our data by sex, urban/rural location and age (10–14, 15–19, 20–24) and randomly selected 10 narratives from each of the 12 resulting strata, oversampling locales if necessary to increase likelihood that 20 stories were selected for each age/sex stratum. In some countries, certain age/sex strata still contained fewer than 20 narratives, hence some country samples have fewer than the maximum 120 narratives (Table 1). Our sampling procedures are described in detail in Winskell et al. (2018). An overall sample of 1,343 texts for the five countries resulted.

**Table 1.** Sample distribution.

	1997	2005	2008	2014	TOTALS
Senegal	86	107	79	67	**339**
Burkina Faso	44	112	100	56	**312**
Nigeria	–	120	93	88	**301**
Kenya	–	88	25	116	**229**
Eswatini	–	72	50	40	**162**
TOTALS	**130**	**499**	**347**	**367**	**1343**

Note: Scriptwriting competition was not carried out in Nigeria, Kenya or Eswatini in 1997, therefore no samples exist for these years.

### Data processing and analysis

Our methodological approach is situated at the intersection of grounded theory (Corbin & Strauss, 2008) and thematic narrative analysis (Riessman, 2008). We combine three primary approaches: (1) analysis of quantifiable characteristics of the narratives; (2) qualitative data analysis, focusing on thematically-related text segments and memoing for emergent themes; and (3) a narrative-based approach, focusing on plot summary and thematic keywords. These triangulating methods were developed to enable cross-national and longitudinal analysis and have three main advantages: they grounded the analysis in three distinct, intersecting dimensions of the data; allowed triangulation; and facilitated the generation and validation of interpretive hypotheses.

1. *Quantifiable characteristics*: We quantified discrete components of each narrative, such as direction of sexual transmission (e.g. male to female, etc.), double-entering them into Qualtrics research software (Qualtrics, Provo, UT). Data were downloaded to Microsoft Excel files and descriptive statistics were calculated.
2. *Qualitative data analysis*: Narratives (overwhelmingly handwritten) submitted in English or French were transcribed and entered verbatim into MAXQDA 12 qualitative data analysis software (VERBI Software, 1989–2016), where they were labelled with reference to a codebook of 54 thematic codes, including ‘fidelity/infidelity/multiple partners’.
3. *Narrative-based approach*: A one-paragraph summary was written for each narrative comprising key elements of plot and message. Summaries were independently double-coded with up to six out of 44 keywords, which included ‘Fidelity/Infidelity’. Discrepancies in coding were resolved through dialogue.

While the purpose of this study is to inform HIV prevention and communication around fidelity, the young authors commonly depicted fidelity in relation to infidelity. We therefore analyze representations of fidelity and infidelity. Our qualitative analysis addresses both narratives coded with the ‘Fidelity/infidelity’ keyword, and narratives that included thematically coded ‘fidelity/infidelity/multiple partners’ segments. We refer to representations of sexual exclusivity within monogamous or polygynous relationships as ‘fidelity’ and multiple concurrent sexual relationships involving at least one stable partner as ‘infidelity’. Existing thematic coding across a range of deductive (e.g. ‘condom’) and inductive themes (e.g. ‘Unmarried romantic relationships & values’) was complemented with fine-coding specific to the fidelity/infidelity themes. Examples of fine codes include ‘peer and parental influence’ and ‘trust/honesty’.

Across the four time points, 239 narratives had fidelity/infidelity-related quantitative attributes, keywords, and/or thematic codes. Intersecting quantitative, narrative-based and thematic analysis was conducted on these 239 narratives to describe representations of fidelity and infidelity and compare them cross-nationally and longitudinally.

This study, comprising the secondary analysis of existing data, was approved by [de-identified] Institutional Review Board. We cite the narratives verbatim, with the exception that names have been changed. Country names are abbreviated as follows: SN – Senegal; BF – Burkina Faso; NG – Nigeria; KY – Kenya; and SZ – Eswatini. Excerpts are identified by the country, contest year, sex, age, and geographic location of the author. For example, an excerpt followed by ‘(SZ 2014, F 21 U)’ comes from a 21-year-old female participant from an urban area of Eswatini, who participated in the 2014 Global Dialogues contest.

This study has limitations. As contest participants self-select, the data are not representative of the youth populations; participants may be better educated, and more knowledgeable and motivated about HIV than the general youth population. As a product of the same contest mechanism, however, these biases are likely to be consistent across the five countries hence the country samples, though not representative, are comparable for our purposes. We have little demographic information about individual participants other than their sex, age, country of origin, and place of residence. The data are embedded within cultural norms of performance, discourse and persuasion (Farmer & Good, 1991), which may be informed by rhetorical considerations specific to the scriptwriting competition, reflecting the young authors’ motivation, for example, to tell what they consider to be a good story and thereby win the contest. In light of the circumstances in which the texts were written, it is impossible to know which depict lived versus imagined experience: we treat all as representations providing insights into sense-making and related cultural resources around fidelity and infidelity.

## Results

### Overview of fidelity and infidelity

#### Quantifiable characteristics of narratives

The themes of fidelity and infidelity are present in 239, or 18%, of the narratives contributed in 1997, 2005, 2008 and 2014. These narratives were contributed by equal numbers of male and female authors with an average age of 17.0 years (standard deviation = 3.4 years). This reflects author demographics of the larger sample from which narratives were drawn (Winskell et al., 2020). The overall proportion of fidelity and/or infidelity within the narrative sample dipped slightly after 1997 but has remained consistent since then, albeit in different proportions across the countries (Table 2). Fidelity and infidelity as central themes, as measured by keywords, do not vary substantially across the years, accounting for 13% of the sample in 1997, 12% in 2005, 9% in 2008 and 11% in 2014.

**Table 2.** Number and proportion of narratives depicting fidelity or infidelity, as measured by thematic codes.

	SN	BF	NG	KY	SW	Total
1997	18 (21%)	12 (27%)	–	–	–	30 (23%)
2005	21 (20%)	14 (12%)	23 (19%)	18 (21%)	12 (17%)	88 (18%)
2008	14 (18%)	15 (15%)	19 (20%)	5 (20%)	7 (14%)	60 (17%)
2014	8 (12%)	11 (20%)	9 (10%)	23 (20%)	10 (25%)	61 (16%)
Total	61 (18%)	52 (16%)	51 (17%)	46 (20%)	29 (18%)	239 (18%)

Fidelity, or the intention and practice of sexual exclusivity in a dating or marriage relationship, is referenced in 131 (roughly 10%) narratives in the sample. However, most of these references are brief calls to ‘be faithful’ that occur in narrator messages at the beginning or end of the texts while the plotlines do not incorporate fidelity. Only 47 (3.5%) narratives in the sample include depictions of at least one character practicing fidelity. In these 47 narratives, fidelity is not represented as an effective HIV prevention strategy. Three out of four of those practicing fidelity become infected; these infections occur primarily as a result of their partner’s inability or refusal to remain sexually exclusive or due to infection via blood. In the few narratives that describe mutual fidelity (*N* = 10), almost all depict one partner becoming infected without realising prior to entering into the mutually exclusive relationship, placing their partner at risk.

Representations of infidelity are much more common in the sample. 220 narratives, or 16% of all 1,343 narratives, include representations of infidelity within unmarried and married relationships. While most infidelity is portrayed within a couple, 15 of these narratives (contributed by Burkinabe, Kenyan and Swati authors) depict infidelity within polygynous relationships. At times, infidelity occurs with multiple long-term concurrent partners (such as a husband with a wife and mistress), but more often the extent of time overlap is not described. Infidelity leads to HIV infection 9 out of 10 times in the narratives contributed on this theme; one-time sexual encounters are represented as carrying equal risk for HIV infection as longer relationships.

Male infidelity is represented twice as often as female infidelity, however this difference decreases over time. In 1997, the ratio of representations of male infidelity to female infidelity is 1–3; 2–9 in 2005; 3–5 in 2008; and 1–1 in 2014. Authors represent male infidelity as facilitated most often by sexual access. Male characters either seek out extra relationships in bars, brothels and similar settings, or become aroused by the sexual availability of other (typically younger) female characters such as house helps, secretaries, and friends of their wives or girlfriends. Often, sexual availability leads to arousal that becomes difficult to control: ‘Her breast and buttocks were captivating to behold. Chief Okoro was carried away. He could not control his emotions. He became intensely erotic’ (NG 2008, M 24 R). Travel and migration facilitate access to new sexual partners beyond the eyes of wives or girlfriends. Authors depict male characters rejecting any perceived risk of HIV transmission, either by denying the existence of HIV or ignoring their own vulnerability.

Representations of female characters’ infidelity become more common over time, increasing steadily from 1 in 8 narratives that include fidelity/infidelity themes in 1997 to 2 in 5 in 2014. Female infidelity is most often linked to transactional sex. In the earlier years, this transactional sex is primarily driven by poverty and the need to provide for their families. Over time, authors increasingly represent female characters as motivated by a desire for wealth or status symbols, such as clothes and mobile phones. For example, one narrative describes Amanda, a poor, beautiful married woman who attracts the attention of a rich businessman. This man, Alex, offered her gifts to seduce her into an affair:

Amanda didn’t even think twice of the gifts she took them because all her life she wished for fancy clothes and shoes … She started sleeping outside and she also despised the husband. Alex gave her all she wanted, money, fancy clothes and cars. (KY 2014, F 17 R)

Authors from all countries more harshly condemn female characters that take on multiple partners out of desire for gifts or wealth, rather than poverty, for their materialism and misplaced values. Blame is communicated via narrator commentary, dialogue between characters, or via narrative plotline, especially humiliating deaths. In Amanda’s case, after describing how Amanda loses her good looks, job and loving husband, the author concludes the narrative: ‘Indeed pride comes before a fall. She learnt it the hard way.’

#### Longitudinal thematic distinctions

Certain longitudinal developments emerge in the treatment of fidelity and infidelity. In addition to the increase in representations of female infidelity over time, later years – particularly 2008 and 2014 – reflect an increased call to support characters who have been infected via infidelity and to prevent HIV transmission within a serodiscordant couple. This is made possible via increased references to antiretroviral therapy (ART) and is often reinforced by medical personnel, family and friends. The infidelity is rarely addressed, although isolated narratives describe the couples as mutually faithful post-infection. Apart from these exceptions, representations of fidelity and infidelity in relation to HIV infection remain relatively consistent across the time points.

#### Cross-national thematic distinctions

Although there is no substantial difference in prevalence of fidelity/infidelity narratives across the countries, several cross-national thematic differences were observed in the data. Senegalese narratives are characterised by male travel and migration as a driver of infidelity; labor-driven migration facilitates an increase in wealth and subsequent access to sexual partners. This newfound wealth is often represented as morally corrupting, in that male characters eschew their responsibilities to their families in favour of sexual liaisons and, ultimately, bring HIV into the household.

Burkinabe authors have the lowest proportion of representations of fidelity in their narratives and rarely imply that sexual fidelity is an expectation of a relationship, evident by the measured reaction of characters (primarily female) when infidelity is revealed. Rather than decrying the immorality of infidelity, Burkinabe authors criticise placing one’s partner and/or family at risk of HIV. For example, Jacob travels for work and has sex with prostitutes. Aware of the risks of HIV, he gets tested and after testing negative, ‘refuses to change his ways’ (BF 1997, M 19 U). Through his continued infidelity he infects his wife: ‘Lucy, (Jacob’s) wife, was an innocent victim of AIDS. She was infected by her unfaithful husband.’ After he dies, she is left wondering ‘if she should cherish or hate his memory for having passed (her) the sickness.’ Arguments of morality do not centre around mutual fidelity but rather on failing to protect others from HIV infection.

The Nigerian sample includes the highest proportion of representations of relationships characterised by romance and melodrama, whereby fidelity is a relationship requirement and infidelity a moral failing. These narratives promote abstinence until marriage and fidelity within marriage, commonly articulated as avoiding ‘sexual immorality’ (NG 2005, F 14 U). In contrast to other countries in the sample, female characters in Nigerian narratives across all time points are represented as having multiple partners as often as male characters. Female characters are primarily motivated by a desire for wealth and gifts, rather than forced by poverty, and consequently Nigeria has the highest proportion of narratives featuring a ‘sugar daddy’.

The Kenyan sample is divided in terms of depictions of fidelity and blame for infidelity. Like Nigerian authors, many Kenyan authors describe infidelity as a moral failure to uphold expectations of sexual exclusivity, citing the ABCs as behavioural prevention strategies in moralistic commentary intended to denounce the unfaithful partner. Depictions of the vulnerability of faithful female characters underscore the pitfalls and limitations to fidelity as a primary prevention method. In a narrative titled ‘Infidelity that dashed all dreams’, Jacky is an ‘innocent wife’ who becomes infected due to her husband’s ‘illicit unprotected sex’ with ‘well known red light district frequenters’ (KY 2005, M 14 U). However, other authors depict the vulnerability of male characters to accessible young females: ‘It happened that many a men could not resist this young beauty and as much as the old man was a victim, so were his sons’ (KY 2005, F 21 R). The Kenyan sample includes the highest proportion of HIV entering a family via infidelity as a consequence of multiple partnerships within a household. House helps and maids serve as vectors for HIV in these scenarios.

Swati representations of fidelity range from moralistic commentary depicting fidelity as an ideal to representations of couples pragmatically navigating infidelity using a combination of methods, such as via condoms or couple testing. It is this range of representations that distinguishes the Swati sample. While a portion of Swati authors follow similar patterns seen in Nigerian and the more moralistic Kenyan narratives, others display greater empathy in their depictions of infidelity. For example, one 2008 Swati narrative describes a pregnant wife struggling to tell her husband about her HIV status as it will reveal her infidelity several years earlier. She finally blurts out ‘“I am positive” … Nomcebo cried bitterly as he comforted he was crying also’ (SW 2008, F 17 R). While he is initially upset, he soon gathers himself together, tests negative, and goes on to support his wife. In this narrative, support for an HIV positive partner reflects ongoing love and commitment despite the implied betrayal of infidelity.

### Fidelity and infidelity: key themes

Several key themes arise in the young authors’ representations of fidelity and infidelity: in particular, travel and migration as a facilitator of infidelity; expectations of fidelity in companionate romantic relationships; and communication and promotion of HIV prevention within couples and amongst peers.

#### Travel and migration

Travel and/or migration for economic reasons occurs in roughly 1 out of 4 of the narratives on fidelity and infidelity; almost half of these narratives were contributed by Senegalese authors. The young authors represent travel and migration as facilitators of economic and sexual opportunities, which opens the door for primarily male characters to abandon their family responsibilities and engage in extramarital sex:

This is a young person who had gone to Europe to make a living. Life smiled on him and he began to earn money. He forgot his wife, whom he had left in his country and began to throw himself into the arms of the Europeans. (SN 2008, M 22 U)

At times, authors highlight the economic impact due to the loss of the breadwinner to HIV as further proof of the tragic effects such travel-related infidelity has on a family.

#### Companionate relationships with romantic love

Relationships within the sample span from representations of hierarchical relationships predicated on male control of resources to companionate relationship models. We refer to relationships as ‘companionate’ when they include an emotional connection based on individual characteristics of each partner, romantic love, trust, and/or expectations of sexual exclusivity. Companionate relationships with romantic love often include other indicators of modernity and socioeconomic achievement, such as elaborate weddings, education and career advancement, international travel and modern homes.

Authors highlight expectations of fidelity as central to assumptions of romantic love. This is most often made explicit upon the discovery of infidelity. Depending on the tone of the narrative, reactions to this discovery reflect feelings of betrayal and heartbreak or, in the event of HIV diagnosis, may result in declarations of support and continuing commitment to the relationship. Feelings of betrayal and heartbreak occur primarily in narratives characterised by melodrama that critique the morality of characters based on their sexual behaviour. Heartbreak follows the discovery that commitments of fidelity were violated:

LUCY: Please forgive me … I am a harlot … I have many playboy outside that I enjoy and satisfy my passion with, please don’t refuse me accept …  … OBINNA: Shut up, just shut up there (tears rolled down his cheek) lucy you have the mind to do all this folk behind our marital home. (NG 2014, F 17 R)

Infidelity in these melodramas represents moral failures (in Lucy’s case, she is described in the introduction to the narrative as ‘Obinna wife, who failed him’).

In cases of infidelity within companionate relationships with romantic love, many authors depict female characters expressing initial dismay but overcoming their negative feelings to support their husbands. In contrast, female infidelity within romantic love relationships is most often condemned as indicative of loose sexual morals.

#### Interpersonal communication and HIV prevention

##### Within couples

Representations of interpersonal communication around fidelity, infidelity and HIV prevention include dialogue within couples and amongst peers. Discussions within couples are rarely represented as preventive; rather, conversations most often occur after HIV diagnosis when a couple is tasked with addressing both infidelity and infection.

Only a handful of narratives depict discussions around expectations of mutual fidelity in relationships. Amongst those that do, the couples are younger and in unmarried relationships. In these scenarios, authors place emphasis on the value of abstaining until marriage and remaining faithful within a monogamous relationship: ‘I beg you to do as I did, that is to say, to abstain until we get married … do not betray me and finally I trust you’ (SN 1997, M 19 U). In all of these narratives, expectations of fidelity are not adhered to and both partners get HIV.

##### Combining HIV prevention methods: condoms, counselling and testing and biomedical options

The large majority of fidelity and infidelity narratives do not depict condom use with either primary or secondary partners; rather, characters are most often depicted as having ‘unprotected sex.’ Several unique narratives depict wives and girlfriends initiating conversations about condoms with male partners, promoting condom use as an act of love (SW 2005, F 13 R), protection (BF 2008, F 19 R) and as part of a sexual seduction (KY 2008, F 21 R). All three narratives acknowledge the vulnerability of the female character in the face of her husband or boyfriend’s sexual liaisons and depict different approaches to framing condom promotion in instances of presumed infidelity. No narrative depicts a male character initiating similar conversations about condom use with a female partner.

HIV testing and counselling are far more common in fidelity/infidelity narratives than representations of condoms, occurring in roughly half of narratives with these themes. Scenarios in which couples mutually decide to get tested are rare. More commonly, one partner – almost always the wife or girlfriend – upon observing symptoms or risky behaviour in her male partner or as part of an antenatal checkup, decides to get tested and convinces her partner to also get tested. In a small portion of the narratives, HIV testing and disclosure become a turning point for the couple, with an unfaithful spouse seeing the error of his ways and changing his behaviour. While authors of these narratives underscore the enrichment of the relationship that comes from monogamy, testing rarely leads to attempts to prevent the infection of an HIV-negative partner; it does lead to connection to care and prevention of mother-to-child transmission (PMTCT) in a small portion of narratives, most prominently in 2008.

Representations of biomedical methods for prevention purposes are nonexistent within the fidelity/infidelity narratives. While authors in the later years depict faithful and unfaithful characters diagnosed with HIV being connected to antiretroviral therapy (ART), none cite its preventive effects (treatment-as-prevention). Similarly, no 2014 narrative references other biomedical methods that might provide additional prevention, such as voluntary male medical circumcision (VMMC), or pre- or post-exposure prophylaxis (PEP or PrEP).

##### Amongst peers

Representations of peers supporting and critiquing characters’ decisions to seek sex outside of their relationships provide a forum for debate around the motivations for and pitfalls of fidelity and infidelity. At times, male peers facilitate infidelity by providing a venue for alcohol consumption and male comradery away from wives and girlfriends, while female peers promote infidelity via support for transactional sex.

More commonly, however, peers disseminate cautionary messages urging fidelity to a protagonist who is considering or practicing infidelity. While these peer interactions occur within male and female groups, the majority of debates amongst peers on the merits of fidelity and infidelity feature male characters. These peer messages, most common in Nigerian and Senegalese narratives, draw primarily on notions of protecting oneself and one’s family from HIV. In one Nigerian narrative, a male peer tells his philandering friend: ‘I’m a family man, I don’t want my children to suffer because of me, let me tell u the reason why I doesn’t look in to another woman’s face, I am afraid of this deadly disease called HIV’ (NG 2008, M 22 U). Others depict peers chastising unfaithful characters for failing to uphold their moral obligation to remain monogamous, contrasting the philandering with their own decision to be faithful.

You Modou, I know you’re young and married but do not worry. Sometimes you have to shoot elsewhere, there is nothing serious about it. Otherwise you will lose big.’ Modou gets carried away and reacts instantly … How dare you offend me that way you atheist? Never do that again. And know that I love only my wife. I am faithful and I wish to remain it forever with the grace of God. (SN 1997, M 23 U)

Fidelity and infidelity in these scenarios become a reflection of the personal values of characters. In the case of this narrative, the personal values align with religious mores.

##### Combining HIV prevention methods: condoms

When it becomes apparent that a protagonist is unwilling to remain faithful, peers are the most common source of condom promotion. Despite these peer influences, the majority of protagonists reject their peers’ advice to be sexually exclusive or use condoms with secondary partners, arguing either that HIV is not real or they are not at risk of transmission:

MR. AZIBA: Take time, you have a wife at home has everything that butterfly has.

MR. OBIOMA: Is it your business?

MR. AZIBA: It is not, but remember AIDS is real … My advice is for you to use a condom.

MR. OBIOMA: I want to enjoy it so, I won’t use a condom … I have told you, nothing like [AIDS] exists.

MR AZIBA: Since you won’t listen, I’ll leave you alone. But remember, the stubborn fly follows the corpse to the grave (NG 2008, F 14 U).

## Discussion

These results present social representations of fidelity and infidelity in young Africans’ creative narratives across countries and over time. Fidelity is often promoted as the ideal by narrators, peers and romantic partners, in line with broader discourses around HIV prevention, romantic relationships, familial obligations, and religious and moral imperatives. However, mutual fidelity is rarely modelled by protagonists and in fact is overwhelmingly portrayed as an ineffective strategy to prevent HIV transmission. Representations of infidelity predominate the sample, with female infidelity increasing in later years. Despite efforts to promote fidelity in the different countries, representations of fidelity evolve little over time.

Several important issues were not addressed in our data. First, there is a relative absence of representations of couples making use of other prevention methods, such as couples testing or condoms, and longitudinally there are no shifts in representations that reflect the emergence of biomedical prevention, such as pre-exposure prophylaxis (PrEP), post-exposure prophylaxis (PEP), voluntary male medical circumcision (VMMC) and treatment-as-prevention. This absence was identified in other analyses of representations of prevention over time (Winskell et al., 2020), and points to an important potential gap in awareness of effective biomedical prevention tools. The absence of treatment-as-prevention is particularly worrisome, given its value for minimising transmission between serodiscordant couples, and is in line with findings from other qualitative studies carried out in southern Africa (Bond et al., 2016; Mooney et al., 2017). The absence of representations of more well-known practices such as couples testing and condoms aligns with another key absence – specifically, the absence of representations of couples discussing their expectations for fidelity or how to pragmatically navigate infidelity (e.g. through additional preventive methods). Instead, infidelity is discovered upon HIV diagnosis, often of the faithful female partner.

### Fidelity promotion

These patterns reflect loss-framed messages using fear arousal techniques that highlight the negative outcomes of taking action or of not taking action. Historically, fidelity has often been promoted using loss-framed messages that underscore the danger of infidelity and non-monogamy in the context of HIV; negative outcomes include risks for HIV transmission, to familial wellbeing, to one’s moral character or some combination of these. For example, the 2006 Swati HIV mass media campaign ‘Secret Lovers Kills’ ran an ad depicting two people organising a sexual meetup. The tag line stated: ‘Why kill your family? Secret lovers kill’ (Spina, 2009). Green and Witte (2006) argue contentiously that fear arousal techniques which combine fear with efficacy messages have successfully contributed to shifts in HIV prevalence. They argue that loss-framed messages that use fear increase risk perception of the dangers of a specific action (e.g. having sex with multiple partners could lead to embarrassing yourself and your wife, etc.) which can be overcome when balanced with sufficient support to achieve the desired behaviour.

Critics of these approaches posit that such individual-level fear arousal techniques for fidelity promotion fail to take into account the complexity of the behaviour and the social and economic forces undergirding it. For example, gender and sexual norms influence the degree to which individuals are motivated and able to ensure that they (and their partners) are sexually exclusive. LeClerc-Madlala (2009) identifies larger cultural scripts driving MCPs in sub-Saharan Africa, including assumptions that men, when aroused, will want and need to have sex; that it is women’s responsibility to control male sexuality; and that sex outside of the marital bed is more exciting and fulfilling. We see these cultural scripts in our results, most notably representations of male characters having multiple partners primarily due to sexual arousal when in proximity with female characters other than their wives or girlfriends, such as secretaries, house helps or friends of wives or girlfriends. These cultural scripts exist alongside larger HIV prevention and Christian religious discourses promoting fidelity as a moral imperative; the rise of companionate relationship models has further aligned fidelity with aspirations of modern lifestyles and class attainment (Hirsch et al., 2009). The result, as Parikh (2009) observes, is that men continue to practice infidelity and couples must manage it such that men may still appear to adhere to the masculine requirements of sexual prowess without suffering backlash from the broader community for putting their wives and family at risk of HIV and/or social humiliation. Many narratives in our sample reflect this tension, particularly in the debates between male peers in which friends warn the male protagonist about the dangers of multiple partners, advertences that the protagonists always ignore to their detriment. While the pressure to have multiple partners experienced by male protagonists is internal, it reflects the authors’ engagement with these broader social expectations that men may experience to demonstrate manhood by sexual conquests while simultaneously not embarrassing themselves nor their families via infidelity. However, the framing of these narratives often underscores what male characters lose – specifically, their health and, at times, family and respect from others – when they are caught engaging in infidelity.

Similarly, loss-framed, fear arousal approaches do not address economically-motivated migration which often separates couples for long periods of time (Hirsch et al., 2009). All countries in our sample feature narratives with work-related migration. Separation from spouses and other supportive networks creates both a gap in sexual, emotional and living needs as well as sexual opportunities that are unlikely to be detected. For example, Smith (2007) observed that Nigerian men forced to travel for extended periods of time may take on a girlfriend for both the companionship (emotional and sexual), as well as to meet domestic needs such as cooking and cleaning.

Lastly, fear arousal-based fidelity promotion often lacks skills-based modelling, rendering the efficacy dimension of messages around complex behaviours – such as mutual fidelity – difficult to operationalise in everyday life. In their multi-country evaluation of PEPFAR and USAID-funded HIV prevention programmes for youth, Speizer and Lopez (2007) observed that programmes underscored the dangers of sex outside of marriage while providing didactic information about fidelity within marriage; fidelity messages were often secondary to abstinence-until-marriage promotion. These curricula lacked ‘specific messages and skill-based lessons on partner reduction’ (p. 2), rendering the messages on fidelity difficult to turn into practice, particularly for older youth that were already sexually active. ABC-oriented programmes such as those evaluated by Speizer and Lopez were among those found to be ineffective at reducing HIV in sub-Saharan Africa (Lo et al., 2016).

The relative absence of representations of couples practicing mutual fidelity could, in part, be due to participants’ desire to tell a good story. Cautionary tales (Moore, 2009), or loss-framed narratives that underscore individuals’ responsibility for managing their own risk and that moralise risk in a way that blames individuals for the outcomes of their risky behaviours are perhaps viewed as more engaging and therefore more capable of winning a scriptwriting contest. However, this absence could also point to the gap observed by Speizer and Lopez, in that the young authors may have been exposed most often to education and communication strategies that provided simplistic or didactic messaging around partner reduction or mutual fidelity without incorporating modelling or other supports to facilitate translation into practice. We see didactic messaging most prominently in the narratives that depict a character becoming infected with HIV via infidelity that either start or end with a message promoting fidelity, but otherwise do not depict fidelity or partner reduction in action.

### Implications for HIV communication

These youth-authored creative narratives reflect loss-framed fear arousal techniques in their depictions of fidelity and infidelity. Specifically, across all time points and countries, they depict the dangers of infidelity while pointing to fidelity as the primary HIV prevention method within these scenarios. Very few authors depict a combination of prevention methods or represent couples conversing about their expectations for fidelity, perhaps in part because these representations undermine the message they wish to disseminate regarding the morality of an unfaithful character and, by extension, a person living with HIV. This points to the potential of fear arousal techniques to reinforce stigmatising beliefs and the need to incorporate stigma reduction and skills-building efforts into HIV risk and prevention communication. In the case of the ‘Secret Lovers Kill’ campaign, the ad only ran two weeks due to backlash from Swati citizens and HIV advocacy groups who argued that the campaign branded people living with HIV, and particularly women, as ‘secret lovers’ intent on transmitting HIV throughout society (Spina, 2009).

Strategies that leverage narrative formats such as entertainment-education (Singhal, Cody, Rogers, & Sabido, 2004) are better equipped to address stigma while promoting fidelity and/or safer sex practices. Entertainment-education formats provide opportunities for participants to observe alternative relationship models and strategies to navigate conversations with relationship partners. The OneLove campaign mentioned before (Jana, Letsela, Scheepers, & Weiner, 2015) is an example of such an approach. Organisers used entertainment-education via radio, television and print media, along with community-based group activities, to raise awareness of the risks of MCPs, model mutually faithful relationships and promote safer sex practices. Evaluation findings indicate that the campaign influenced norms and behaviours around fidelity and infidelity, condom use and HIV testing (Scheepers, 2013), though the impact may have been lesser for groups with additional risks for HIV, such as couples in which one partner migrates (Mir, 2016).

Community-based activities could incorporate participatory narrative-based formats into prevention efforts, such as activities that facilitate the deconstruction of harmful cultural scripts (Paiva, 2005) around infidelity and MCPs (LeClerc-Madlala, 2009). Common storylines from our sample could be deconstructed in activities such as those that condemn infidelity as immoral while simultaneously depicting MCPs as a component of normative sexual behaviour, especially for male characters. These activities provide opportunities for participants to build skills to navigate sexual risk within relationships, practice discussing expectations of fidelity, non-fidelity and corresponding prevention strategies, as well as challenge harmful gender norms. These activities could form part of a multilevel communication campaign aimed at addressing MCPs such as OneLove, as a component of more general comprehensive sexual education efforts, or could be incorporated into apps and similar interventions disseminated via mobile phones. There is scope to capitalise upon the advantages offered by mobile phone technologies to design and disseminate innovative sexual health education and communication that provide gain-framed messaging, modelling and interactive skills-building around complex behaviours such as partner reduction and mutual fidelity at scale.

However, these approaches cannot be separated from broader issues of gender and sexual norms and structural realities described above, the majority of which place women at greater risk of HIV transmission due to their inability to ensure their partner’s preventive behaviour. Our results point to the need to combine fidelity communication with gender-transformative and HIV stigma reduction approaches.

### Developments since 2014

Between 2000 and 2013, following the elevation of Uganda’s success in reducing HIV incidence and creation of PEPFAR, there was an increase in research and programmatic action to promote fidelity and/or reduce MCPs within HIV prevention efforts. However, in recent years we could find few publications on fidelity or MCPs beyond methodological advancements in sexual network measures (Morris et al., 2014; Sawers & Isaac, 2017) and quantitative studies demonstrating the continued practice of MCPs (Lukhele et al., 2016; Muchiri, Odimegwu, Banda, Ntoimo, & Adedini, 2017). This perhaps reflects a shift away from fidelity promotion due to the evidence against abstinence-only and fidelity until marriage approaches, or could be an indicator of the broader shift in prevention funding to biomedical prevention (Kippax & Stephenson, 2016). The few qualitative studies with data collected after 2014 provide insight into other forms of sense-making around fidelity with which to juxtapose our findings and point to areas of future research and intervention.

Nalukwago, Alaii, Borne, Bukuluki, and Crutzen (2018) describe Ugandan adolescents’ ambivalence around multiple partners and fidelity. Their adolescent participants underscored the immorality of multiple sexual partners while acknowledging young people’s lack of skills to converse with partners around risks and navigate sexual situations. This confirms our overall findings and recommendations. While programmatic stakeholder participants in the Nalukwago et al.’s study perceived adolescents to lack knowledge of the risks posed by multiple partners, our findings reflect an elevated risk perception of multiple partners that spans country and time point samples. This discrepancy points to the importance of ongoing ethnographic surveillance of youth sense-making around HIV risk and the social drivers of risk in order to inform a response grounded in youth needs.

Manyaapelo et al. (2019) and Swartz, Colvin, and Harrison (2016) draw attention to the male and female experiences with and sense-making around multiple concurrent relationships in South Africa. Manyaapelo et al.’s male participants describe the pressure amongst male peers to demonstrate their masculinity via sexual prowess. Obtaining and sustaining multiple concurrent relationships was understood as arising out of a physiological need for sex. Representations of male peers in our study more often depict male peers promoting mutual fidelity and other preventive methods to resistant male protagonists. This most likely reflects the ways in which the young authors draw on broader discourses available to them to depict the debates around a behaviour they believe to be socially desirable while acknowledging the competing social norms on men to be promiscuous.

Swartz et al. describe the emotional and material dimensions of young women’s MCPs. Their young female participants described maintaining multiple concurrent relationships with men with whom they shared an emotional connection that was, at times, demonstrated and strengthened by financial and material support. The authors in our sample rarely depicted an emotional dimension to female infidelity. Rather, they described female characters motivated by financial necessity or the desire for material gain and associated improved social status, with female characters motivated by the latter reasons blamed more harshly than the former. This reflects findings from other literature around transactional sex (Fielding-Miller et al., 2016; Stoebenau, Heise, Wamoyi, & Bobrova, 2016) that points to the increased stigma towards women that engage in transactional sex in order to obtain modern goods and social status advancement rather than out of financial deprivation. Most notably amongst the more stigmatising narratives in Nigeria and Kenya, the relative absence of representations of the emotional connections and attractions that lead to infidelity and MCPs could be a reflection of these young authors’ desire to tell a moralistic story in which infidelity is denounced as a pathway for HIV infection. Given the value placed on romantic love in representations of fidelity, depicting romantic attachments between unfaithful female characters and their lovers could enhance empathy and complicate the link these authors are communicating between promiscuous women and HIV.

## Conclusion

Results from this cross-national and longitudinal analysis reveal the different ways in which young authors depict fidelity as a way to prevent HIV. A large portion of the representations reflect loss-framed messaging that underscore the dangers of infidelity, reinforce a moral hierarchy between fidelity and infidelity, and perpetuate HIV stigma. Despite the challenges associated with addressing fidelity in particular and partner reduction more generally, the epidemiological evidence pointing to the importance of MCPs in contributing to the HIV epidemic implies that this remains a valuable behavioural prevention strategy. Rather than addressing fidelity alone or only in conjunction with abstinence and condoms, partner reduction can form part of comprehensive sexuality education or combination prevention efforts that promote a menu of preventive practices that can be put into practice in order to minimise HIV risk associated with MCPs. Based on our analysis, we recommend incorporating narrative-based approaches to deconstruct harmful cultural scripts that reinforce unequal sexual and gender norms and model alternative relationship and behavioural norms and provide participatory skills-building opportunities in which young people may practice conversing with sexual partners about relationship expectations, combining HIV prevention methods and navigating sexual risk. Above all, gender-transformative and stigma reduction approaches that engage both men and women must undergird any efforts to promote fidelity or partner reduction and address MCPs within the context of HIV prevention.
